# *Namdinator* – automatic molecular dynamics flexible fitting of structural models into cryo-EM and crystallography experimental maps

**DOI:** 10.1107/S2052252519007619

**Published:** 2019-06-27

**Authors:** Rune Thomas Kidmose, Jonathan Juhl, Poul Nissen, Thomas Boesen, Jesper Lykkegaard Karlsen, Bjørn Panyella Pedersen

**Affiliations:** a Centre for Structural Biology, Department of Molecular Biology and Genetics, Aarhus University, Gustav Wieds Vej 10C, Aarhus, DK-8000, Denmark

**Keywords:** molecular dynamics flexible fitting, MDFF, cryo-EM, crystallography, molecular dynamics, model-fitting, automation, web services, flexible fitting

## Abstract

Model building into experimental maps is a key element of structural biology, but can be both time consuming and error prone for low-resolution maps. Here we present *Namdinator*, an easy-to-use tool that enables the user to run a molecular dynamics flexible fitting simulation followed by real-space refinement in an automated manner through a pipeline system. *Namdinator* will modify an atomic model to fit within cryo-EM or crystallography density maps, and can be used advantageously for both the initial fitting of models, and for a geometrical optimization step to correct outliers, clashes and other model problems. We have benchmarked *Namdinator* against 39 deposited cryo-EM models and maps, and observe model improvements in 34 of these cases (87%). Clashes between atoms were reduced, and the model-to-map fit and overall model geometry were improved, in several cases substantially. We show that *Namdinator* is able to model large-scale conformational changes compared to the starting model. *Namdinator* is a fast and easy tool for structural model builders at all skill levels. *Namdinator* is available as a web service (https://namdinator.au.dk), or it can be run locally as a command-line tool.

## Abstract 2

A pipeline tool called *Namdinator* is presented that enables the user to run a molecular dynamics flexible fitting (MDFF) simulation in a fully automated manner, online or locally. This provides a fast and easy way to create suitable initial models for both cryo-EM and crystallography, and help fix errors in the final steps of model building.

## 1. Introduction   

In recent years, major technical advances in the cryo-EM field have resulted in an increasing number of cryo-EM density maps being deposited (Kühlbrandt, 2014 ▸; Subramaniam, 2019 ▸). With the growing number of maps there is an increasing demand for better and easier methods for fitting atomic models. An analysis of models fitted to cryo-EM maps has previously indicated the presence of notable problems in almost all of the selected structures (Wlodawer *et al.*, 2017 ▸). In 2008, the powerful molecular dynamics flexible fitting (MDFF) method was presented. MDFF can fit a model in a flexible manner into a cryo-EM density map using molecular dynamic simulations (Trabuco *et al.*, 2008 ▸). Implementing MDFF as a standard tool for model building involves a steep learning curve, including preparing and restraining an input model for molecular dynamics simulations, converting EM or crystallographic maps to a potential field, and finally setting up and running the simulations. After a molecular dynamics run, real-space refinement should be carried out to re-adjust the model to optimize geometric constraints. *Namdinator* was developed as a user-friendly automated pipeline to assist with all of these steps.

Through testing and benchmarking we demonstrate that we can obtain excellent MDFF fits of models to cryo-EM maps with no manual intervention by using *Namdinator*. *Namdinator* does not only optimize the fit to the map, but it also improves the quality and geometry of the fitted model through an automatic round of real-space refinement in *Phenix* after the MDFF fit. As such, *Namdinator* assists and speeds up all types of model building and improves the quality of the final model while ensuring a good fit to the observed density.

## 2. Experimental   

### 2.1. *Namdinator* framework   

**Figure 1:**
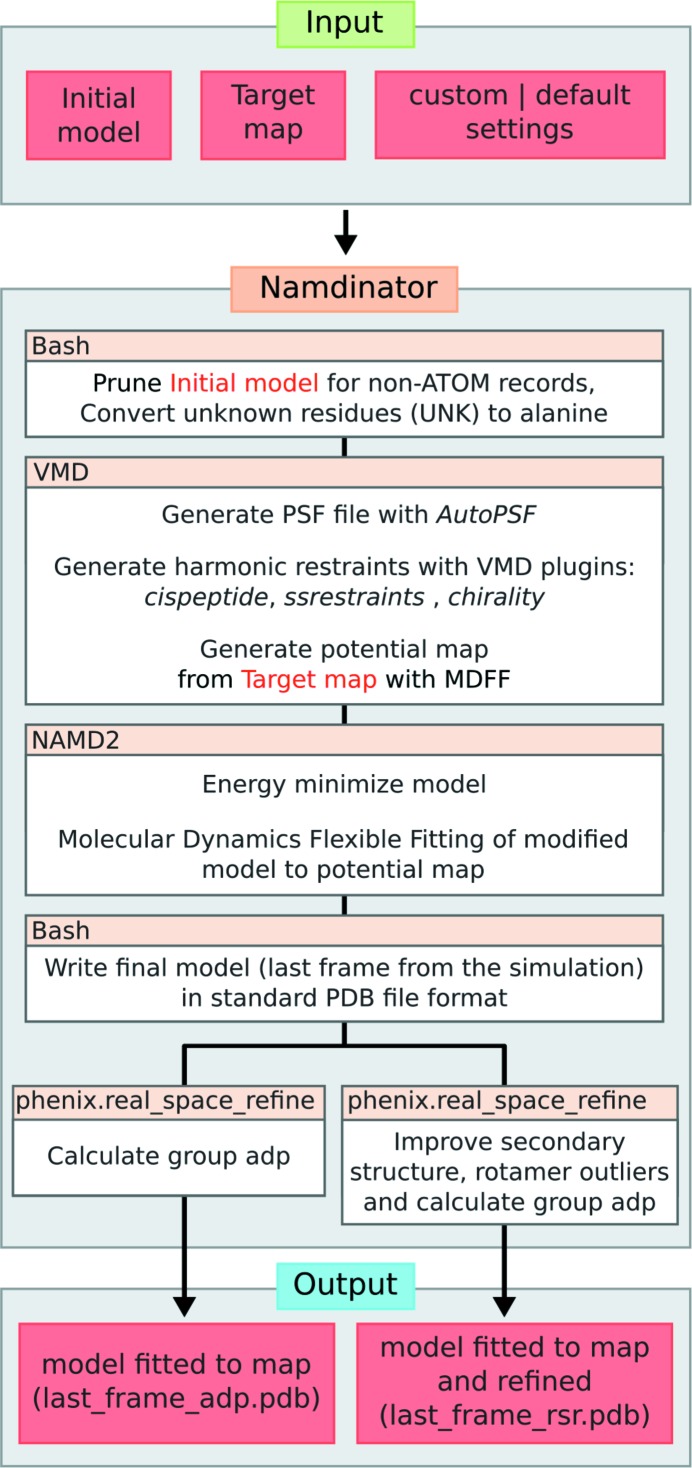
Flowchart for *Namdinator*. As input, the user must provide an initial model and a target map with its corresponding resolution. A number of settings can be set or default values can be used. *Namdinator* then modifies the initial model and runs a *NAMD*2 simulation using MDFF. After the run, *phenix.real_space_refine* will be run on the output to either calculate group ADP only or to perform a real-space refinement and group ADP calculation. The latter, in particular, will help improve Ramachandran plot outliers (backbone/secondary structure improvements), and rotamer outliers.

A flowchart for *Namdinator* is presented in Fig. 1 ▸. To run *Namdinator*, a model in standard Protein Data Bank (PDB) file format and a map are needed. The map can be a cryo-EM density map or a crystallographic map in either mrc or CCP4 format, and it should be in space group *P*1 (default for EM maps). In *Namdinator* the input map is converted to a potential map by MDFF for use with *NAMD2* (Phillips *et al.*, 2005 ▸), but no modification (scaling, filtering, masking) is performed. The initial model can be a homology model or a model of the target protein in a different state/conformation if desired. The model should be roughly docked into the map before using *Namdinator* to ensure that as much of the model as possible is inside the density in the approximate orientation before execution. The necessary accuracy of this preliminary fitting will vary from case to case, and can be tested empirically if necessary. Typically, initial docking can be performed manually or by rigid-body-fitting programs such as *COLORES* (Chacón & Wriggers, 2002 ▸) or *Chimera* (Pettersen *et al.*, 2004 ▸). *Namdinator* does not implement any symmetry constraints on the input model during the run. Within *Namdinator* the input PDB file is prepared for the MDFF simulation using the *VMD* plugins *MDFF* and *AutoPSF* (Humphrey *et al.*, 1996 ▸). *AutoPSF* uses an internal set of standard settings and topology files to generate dynamics-ready PDB and protein structure file (PSF) files. *AutoPSF* will automatically add hydrogens and missing atoms to the model and apply standard MD modifications (patches), *e.g.* for disulfide bridges, termini residues *etc*. While *AutoPSF* allows for a streamlined process, it is prone to failure when encountering non-standard atoms in a PDB file. Therefore, all non-ATOM records are removed, meaning that metal ions, water molecules, ligands *etc*. will not be included in the simulation or in the output PDB files, and must be manually reinserted after the run if so required. All residues designated UNK within the input PDB file are converted to alanine residues. If the starting model is pruned for side chains but maintains sequence information, a full atomic model is generated and used during the simulation, which means that the output will also be a full atomic model. Note that in many cases the deposition of a full atomic model is not suitable, and the user will have to prune the model after the run.

During the MDFF simulation in *NAMD2*, harmonic restraints are applied to the input model in order to prevent over-fitting and structural distortion, and to preserve the stereochemical quality of the model. The simulation is run using the CHARMM36 force field for all-atom systems in a vacuum. *In vacuo* MDFF simulation leads to a substantial speeding up (up to sevenfold) of calculation time compared with generalized born implicit solvent (GBIS) MDFF simulations (Tanner *et al.*, 2011 ▸). *Namdinator* also supports GBIS simulations, which do yield marginally better results judged from the quality of the output models and obtained fit, but at the cost of a substantial increase in running time. After the MDFF simulation, the last frame of the resulting trajectory is exported as a PDB file, hydrogen atoms are deleted and standard PDB atom- and residue-naming format is enforced. This PDB file is then processed in two parallel setups through *phenix.real_space_refine*. In the first, only group atomic displacement factors (ADP) are calculated, and the user can then directly use this output model. In the second setup, the model will both have ADPs calculated and be refined in real space (with the following settings: secondary structure, rotamer, Ramachandran and Cβ deviation restraints, global minimization, group ADP refinement, 5 macro cycles) (Afonine *et al.*, 2013 ▸). We find that this second setup is highly beneficial for cryo-EM models in order to reduce the number of rotamer and Ramachandran plot outliers as well as Cβ deviations, as these parameters are not restrained during simulation. Crystallographic models, being subject to further crystallographic refinement downstream, do not require this step. The final step in both cases is the removal of the *AutoPSF*-generated hydrogen atoms from the model.

### 2.2. Output validation in *Namdinator*   

*Namdinator* performs a validation check of both the input PDB file [after removal of HETATM, conversion of UNK to alanine, and a group atomic displacement parameters (ADP) calculation] and the output PDB file from the simulation and from the *phenix.real_space_refine* run. For validation, *Namdinator* uses the programs *phenix.ramalyse*, *phenix.clashscore*, *phenix.rotalyse* and *phenix.cbetadev* (Adams *et al.*, 2010 ▸). A summary of the validation metrics for each PDB file is displayed in a table at the end of the simulation. Additionally, the *RosettaCommons* package is used to score the PDB files both as whole models and for individual residues (Alford *et al.*, 2017 ▸). The *Rosetta* scores for the models are included with the *Phenix* validation metrics, whereas the top ten flagged residues in each model are reported in a separate table at the end of the run and can readily be assessed for atom clashes *etc*. Together the two tables provide the user with a convenient overview with which to analyze and compare the models. To evaluate how the models fit to the input map, the Pearson correlation coefficients (CC) are calculated between a simulated density map for the model and the experimental input density map. The CC is calculated using both the *MDFF* plugin in *VMD* to obtain a global CC and *phenix.map_model_cc* to obtain a local CC (called CC_mask) around the model (Stone *et al.*, 2014 ▸; Afonine *et al.*, 2018 ▸). For the remainder of this article we will focus on the CC_mask values. The web-service can load the input as well as the output models in an online 3D viewer (*NGL* viewer) (Rose *et al.*, 2016 ▸) together with the target map, for rapid and user-friendly manual inspection of the result.

### 2.3. *Namdinator* system setup   

*Namdinator*’s current third-party software requirements are *VMD* v.1.93 with the following plugins: *MDFF* v.0.5, *ssrestraints* v.1.1, *cispeptides* v.1.3, *chirality* v.1.3, *Autopsf* v.1.6 and *multiplot* v.1.7. All simulations are run within the *CUDA* optimized version of *NAMD2* (2.12) (Phillips *et al.*, 2005 ▸) using the CHARMM36 force field (Huang & MacKerell, 2013 ▸) for all-atom systems in a vacuum. MDFF applies default parameters [step size, 1 fs; force scaling (*G* scale), 0.3 kcal mol^−1^ (adjustable); temperature 300 K (adjustable) using Langevin thermostat coupled to all non-hydrogen atoms with a damping coefficient of 5 ps^−1^; bonded interactions calculated every 1 fs, nonbonded interactions (cutoff 10 Å) calculated every 2 fs (Trabuco *et al.*, 2009 ▸)]. *G* scale is a measure of how hard the target map pulls on the starting model. Temperature controls how easy it will be for atoms to move. For difficult cases it can be beneficial to adjust one or both of these parameters from their default, as well as increasing the number of simulation steps.

*Namdinator* further depends on the *Phenix* package for group ADP and real-space refinement and validation, together with the *RosettaCommons* software package. *phenix.real_space_refine* requires the presence of a CRYST1 record in the header of the input PDB file. If no such record is present, *Namdinator* will append a standard CRYST1 record in space group *P*1. During the *phenix.real_space_refine* run, the unit-cell dimensions will be updated with that of the box size of the input map. By default, *Namdinator* uses GPU accelerated calculation within *NAMD2*, therefore a graphics card supporting *CUDA* v.6.0 or later is recommended for local installations. Additionally, as *NAMD2* does not offload all of the calculations to the GPU, a high-powered multi-core CPU is recommended. 16 GB of memory will enable models containing up to ∼20 000 residues and their corresponding maps to run on local installations. A description of the specific flags that control *Namdinator* are listed in the command-line version of the program. The web-service will allow the user to run *Namdinator* and adjust the above settings within reasonable predefined ranges. Using default settings, the run time on the webserver is approximately 1 min per 15 kDa model, and in general runs will complete within 1 h on a normal workstation environment.

## 3. Results and discussion   

### 3.1. Benchmarking using cryo-EM models and maps   

To benchmark *Namdinator*, 39 randomly selected map and model pairs deposited to the Electron Microscopy Data Bank (EMDB) and PDB were run through *Namdinator* using the resolution stated on their EMDB entry pages. A total of five of the entries were manually modified, specifically to contain unique single-character case-insensitive chain IDs (5gw5 and 5h64), to have only positive residue numbering (6b44), to reduce a multi-model entry to a single model (5nd7), and to expand the model into the full biological unit (3j9c). No atom coordinates were altered before use as input for *Namdinator* and no maps were filtered or modified in any manner. All test cases were run using default *Namdinator* settings. To simplify the comparison of input to output we have focused on three global key statistics. Cross correlation (to gauge the fit between model and map), number of clashes (to gauge the chemical environment of the individual atoms of the models), and Ramachandran plot outliers (to gauge overall protein geometry).

**Figure 2:**
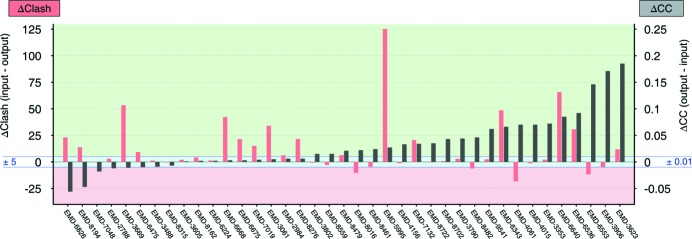
Benchmark of *Namdinator* with 39 deposited cryo-EM structures and maps. Plot of global quality parameters; Δclash score (red bars) and ΔCC (black bars). The individual EMDB entry ID’s are listed along the *x* axis. The delta clash score is calculated by subtracting the clash score of the final output model from *Namdinator* (last_frame_rsr.pdb), from the clash score of the input model (the deposited model). The delta CC value is calculated by subtracting the cross-correlation coefficient between input model and map from the cross-correlation coefficient between final model and map (both from CC_mask in *phenix.model_map_cc*). In both cases a positive value indicates that the output from *Namdinator* was improved compared with the input. The green shading indicates model improvement through *Namdinator*, the pink shading indicates model deterioration, and the blue shading a comparable quality model (±5 clash or ±0.01 CC).

Overall, *Namdinator* improved the test models (Fig. 2 ▸, Supplementary Fig. 1 and Supplementary Table 1). The CC improved in 22 out of the 39 test cases. It was unchanged in 11 cases (within ±1%), and deteriorated in 6 cases. The clash score was improved in 17 cases, 18 cases had a similar clash score (within ±5) and in 4 cases the clash score deteriorated. The number of Ramachandran outliers was reduced in 23 of the cases, identical in 12 of the cases and increased in 4 test cases.

In summary, of the 39 test cases, 34 were improved, some substantially, on at least one of the three global key statistics. In the majority of test cases where one or two of the three key scores deteriorated after *Namdinator*, the starting models were not full-atom models. As *Namdinator* converts the input to full atom models, the deterioration in the majority of these cases can be attributed to this conversion, since generally it is harder to fit a full-atom model into a map while maintaining and optimizing the geometry, compared with a polyalanine model for example. The five cases where all three global key statistics did not improve (6b44, 5ni1, 5sy1, 5n9y, 3j9c) represented relatively high resolution (3.9–2.9 Å) full-atom models, where we would expect *Namdinator* to have the least impact. Thus, evaluated on global statistics, *Namdinator* improved 87% of the test cases. Several models revealed quite extensive improvements, with a significant reduction in the number of severe backbone clashes and unnatural and strained conformations. These are model problems that can also be addressed manually, but clearly an automated method such as *Namdinator* is of great assistance in creating higher quality models based on objective criteria and global targets.

**Figure 3:**
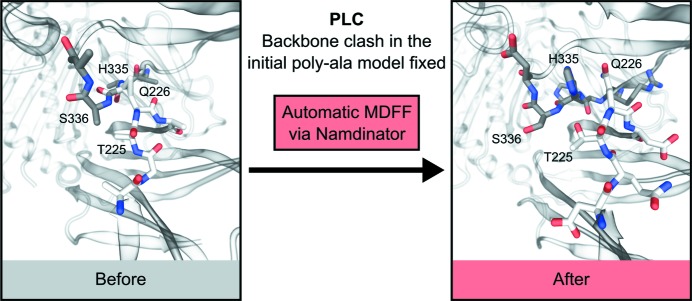
Example of local improvements through *Namdinator*. A severe backbone clash was present in the deposited polyAla model of the peptide loading complex PLC (6eny), between residue Gln226 in chain F and His335 in chain C. *Namdinator* fixed this clash, and by looking at the *Namdinator* log file it was simple to identify the problematic regions in the input model.

The use of global statistics to evaluate quality can hide pronounced local improvements. Visual inspection showed further quality improvements that are not easily quantified. One example was the peptide loading complex (PLC), using the map EMDB-3906 and its associated model 6eny, which was deposited as a polyalanine model (Blees *et al.*, 2017 ▸). The initial clash score of 6eny is 12.9, with 33 Ramachandran outliers. The CC between the deposited model and the map is 56.2%. The output model from *Namdinator*, which is now a full atomic model, obtains a clash score of 17.7 with 2 Ramachandran outliers, while the CC increases to 73.6%. Since 6eny is a polyalanine model the input model clashes originate from CB and/or backbone atoms only and indicate severe backbone clashes in the model. By manually inspecting the residues with the highest individual *Rosetta* scores, as listed in the validation table output from *Namdinator*, several backbone clashes were readily identified in the input model and these had been fixed by *Namdinator* (Fig. 3 ▸).

During testing of deposited maps and models, one case was an obvious outlier with a huge CC increase (EMD-3765/5o9g) (Farnung *et al.*, 2017 ▸). Upon inspection, it was observed that the model was systematically shifted in one direction relative to the map. The systematic shift had occurred during the data deposition, without the authors’ knowledge (Farnung, personal communication). *Namdinator* caught this shift and moved the model back into density within the first 1000 simulation steps. This case is omitted from the final list of examples, but it illustrates the use of *Namdinator* as a rigid-body fitting tool even for models that are systematically shifted relative to their density. However, we would generally recommend rough positioning in the map as mentioned earlier.

### 3.2. Large-scale movements and conformational changes   

We tested *Namdinator*’s ability to fit models, when large parts of the starting model were placed outside the target density. MDFF has been used successfully for fitting one conformation of a protein into the map of a different conformation of the same protein, and since *Namdinator* runs MDFF automatically, it should be able to handle this type of scenario with no or minimal intervention.

**Figure 4:**
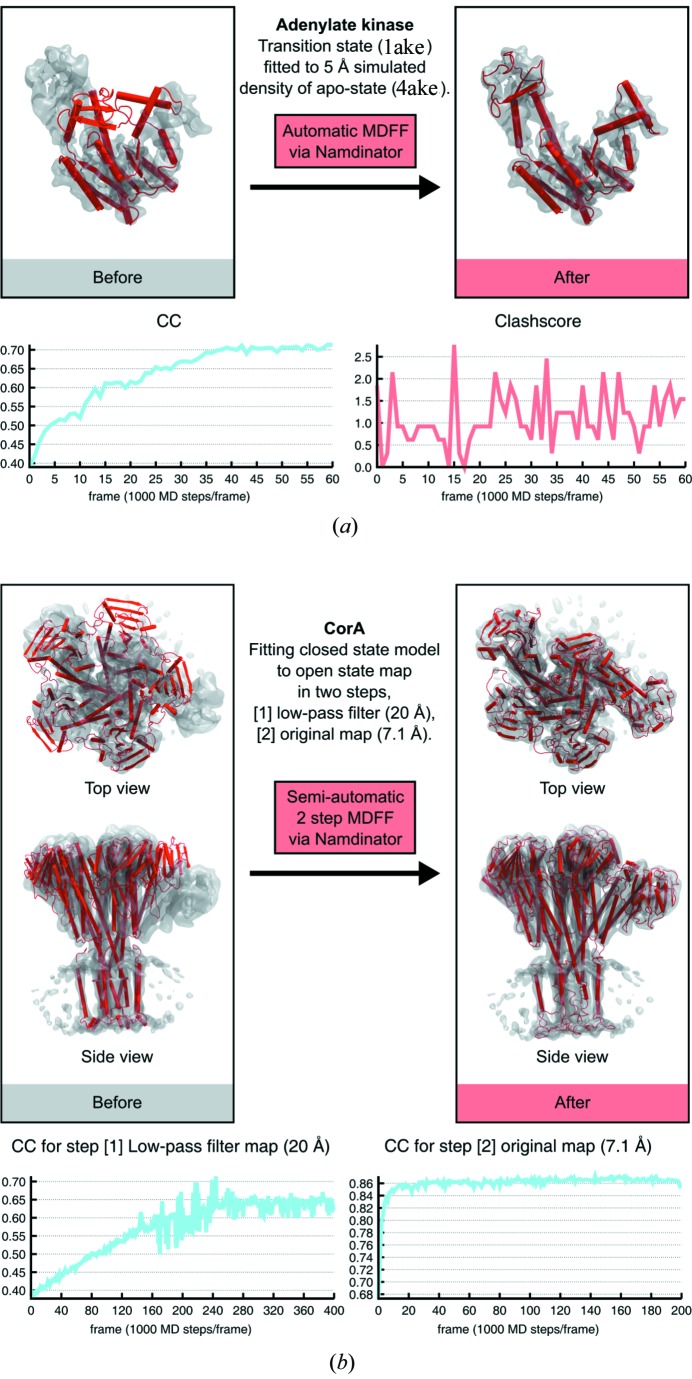
Large-scale movements of models into density. (*a*). Fitting of *E. coli* adenylate kinase transition-state conformation (1ake) into a 5 Å simulated density of the adenylate kinase in the apo-state conformation (4ake) using *Namdinator* with a plot of the CC_mask values and clash score for every 1000th step from *Namdinator*. Default settings were used, except the simulation steps which were increased from 20 000 to 60 000 to reach convergence. (*b*) Fitting the open conformation of the magnesium channel CorA into the density (EMD-6553) of its closed state using *Namdinator*. The initial model (red cartoon) rigid-fitted to EMD-6553 (grey surface), that was used as input for a two-step *Namdinator* procedure. A plot of the CC_mask values for every 1000th step from both sequential *Namdinator* runs are shown.

A classic MDFF test-case used to demonstrate the power of MDFF (Vashisth *et al.*, 2012 ▸) is the fitting of the catalytic transition state of *Escherichia coli* adenylate kinase (1ake) (Lou & Cukier, 2006 ▸) into the apo-state of the adenylate kinase (4ake) (Müller & Schulz, 1992 ▸). We ran this test using *Namdinator* with a 5 Å simulated density of the apo-state. After 60 000 simulation steps a very good fit was obtained and the CC had increased from 39.0% to 71.3%. [Fig. 4(*a*) ▸ and Supplementary Movie 1]. This shows that *Namdinator* can model large movements between conformational states with a noise-free simulated map (derived from a PDB model) as the test case.

*Namdinator* can also handle more challenging cases, such as noisy maps. We used the magnesium channel CorA as a test case (Matthies *et al.*, 2016 ▸), where different conformations of CorA have been determined by cryo-EM including a closed conformation at 3.8 Å and an open conformation at 7.1 Å. During the transition from the closed to the open conformation, CorA changes from being a fivefold symmetric structure to an asymmetric structure where 4 of 5 subunits are being displaced between 10 to 25 Å and inter-subunit contacts undergo hinge-bending motions with up to 35° (Matthies *et al.*, 2016 ▸). To perform the fit, the closed-conformation model (3jcf/EMD-6551) was first rigid-body fitted into the original 7.1 Å cryo-EM density map of the open-state conformation (3jch/EMD-6553) using *COLORES*. A default *Namdinator* setup led to domains and secondary-structure elements becoming stuck at intermediary positions during the MDFF fit. To solve this, the map was low-pass filtered to 20 Å, before being piped into *Namdinator*. Default *Namdinator* settings were used except for the *G* scale which was decreased from the default of 0.3 kcal mol^−1^ to 0.05 kcal mol^−1^ while the number of steps was increased to 400 000 steps. The MDFF output of an initial run with the low-pass filtered map was then used as input for a second *Namdinator* run fitting into the original unfiltered 7.1 Å map where again default settings were used except that the *G* scale was increased to 5 kcal mol^−1^, and the number of steps was increased to 200 000. With this two-step semi-automatic *Namdinator* procedure, MDFF successfully fitted the 3jcf model into the density of the open-state conformation of CorA. The final result was compared with the deposited model for the open state of CorA. The RMSD of the two models was 1.6 Å, *i.e.* a fit within the experimental error of the models at 7.1 Å resolution, but with little manual intervention [Fig. 4 ▸(*b*), Supplementary Movie 2].

## 4. Concluding remarks   

*Namdinator* is an automatic and user-friendly pipeline for flexible fitting and geometrical optimization of a given model to a given map. *Namdinator* runs *AutoPSF*, MDFF via *NAMD2* and *phenix.real_space_refine* with minimal user-input. Running time is fast, generally less than 1 h, and we find that it can efficiently facilitate model building. Importantly, *Namdinator* enables higher quality depositions compared with the current state-of-the-art (Wlodawer *et al.*, 2017 ▸). Exceptions do occur, but as demonstrated here, a default *Namdinator* run will improve model geometry, while maintaining or improving the fit to the map in almost all cases, and for difficult cases minor adjustments of parameters and procedures will support the application.

MDFF has previously been demonstrated to be very powerful for initial fitting of models in both cryo-EM and crystallography (Trabuco *et al.*, 2008 ▸). To ensure a more general use across methodologies, we have prepared the pipeline to also function with crystallographic maps where the same benefits from a fast and easy-to-use pipeline are applicable.

*Namdinator* is a fast way to generate initial plausible models and to correct geometrical errors in the final steps and is a valuable tool in the challenging process of constructing structural models. In the end there is no substitute for human interaction during model building, and *Namdinator* (and similar tools) should not be seen as a replacement for a final and careful manual inspection of a model, but solely as a tool to assist and speed up this process.

*Namdinator* is released under the GNU General Public Licence version 3 (GNU GPLv3) and is freely available as a Linux command-line tool on github (https://github.com/namdinator) or by contacting the corresponding authors. A web-service is available at https://namdinator.au.dk.

## Supplementary Material

Supplemetary Movie 1. DOI: 10.1107/S2052252519007619/eh5002sup1.avi

Supplementary Movie 2. DOI: 10.1107/S2052252519007619/eh5002sup2.avi

Supplementary Figure and Table. DOI: 10.1107/S2052252519007619/eh5002sup3.pdf
