# Supplementary material for: Namdinator – automatic molecular dynamics flexible fitting of structural models into cryo-EM and crystallography experimental maps
Source: IUCrJ. 2019 Jun 27;6(Pt 4):526–31. doi: 10.1107/S2052252519007619 (PMC6608625; doi:10.1107/S2052252519007619)
Supplement: Supplementary file 3 [file m-06-00526-sup3.pdf]

IUCrJ

Volume 6 (2019)

Supporting information for article:

Namdinator - Automatic Molecular Dynamics flexible fitting of structural models into cryo-EM and crystallography experimental maps

Rune Thomas Kidmose, Jonathan Juhl, Poul Nissen, Thomas Boesen, Jesper Lykkegaard Karlsen and Bjørn Panyella Pedersen

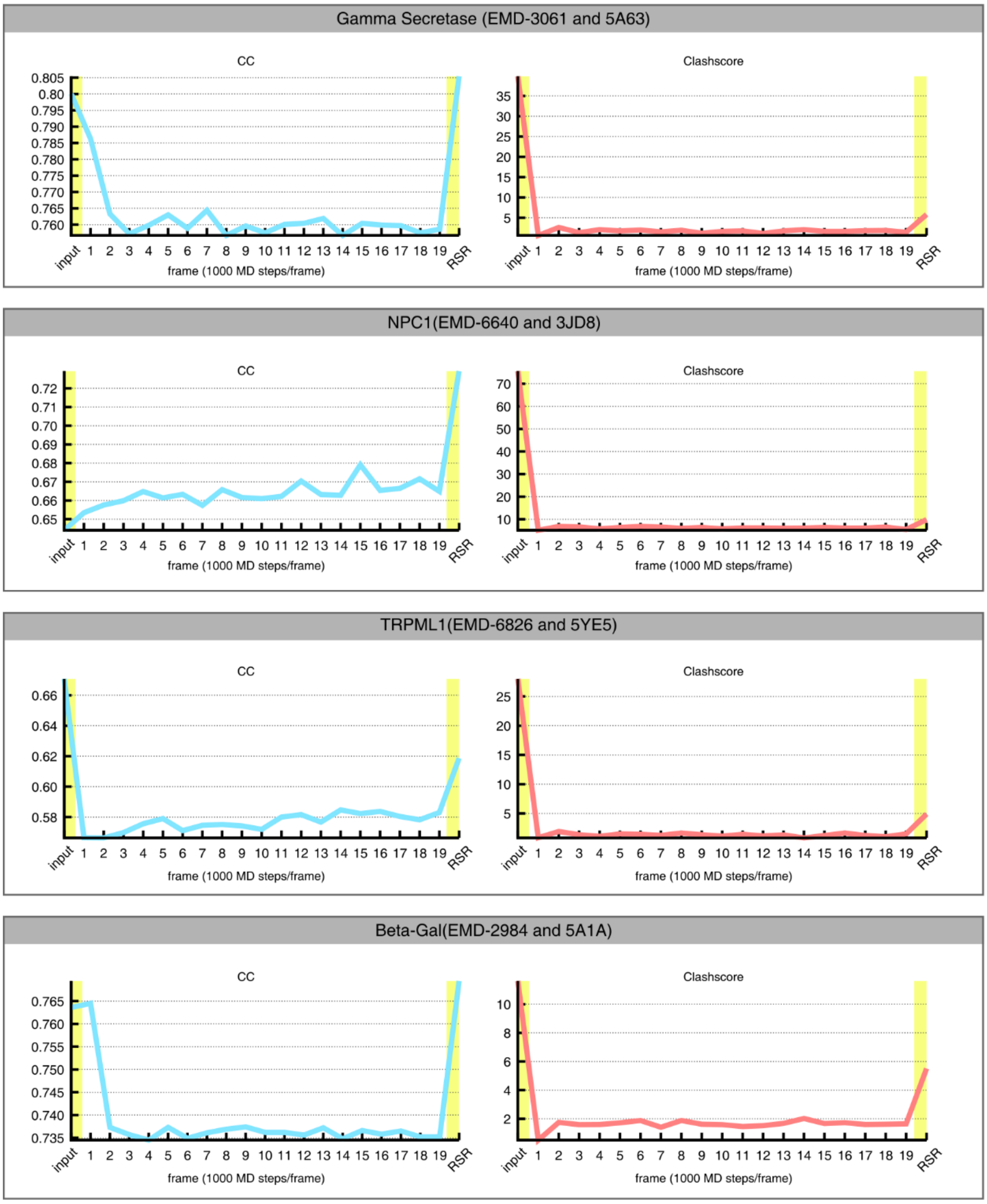

Gamma Secretase (EMD-3061 and 5A63)
CC
0.805
0.80
0.795
0.790
0.785
0.780
0.775
0.770
0.765
0.760
input 1 2 3 4 5 6 7 8 9 10 11 12 13 14 15 16 17 18 19 RSR
frame (1000 MD steps/frame)
Clashscore
35
30
25
20
15
10
5
input 1 2 3 4 5 6 7 8 9 10 11 12 13 14 15 16 17 18 19 RSR
frame (1000 MD steps/frame)
NPC1(EMD-6640 and 3JD8)
CC
0.72
0.71
0.70
0.69
0.68
0.67
0.66
0.65
input 1 2 3 4 5 6 7 8 9 10 11 12 13 14 15 16 17 18 19 RSR
frame (1000 MD steps/frame)
Clashscore
70
60
50
40
30
20
10
input 1 2 3 4 5 6 7 8 9 10 11 12 13 14 15 16 17 18 19 RSR
frame (1000 MD steps/frame)
TRPML1(EMD-6826 and 5YE5)
CC
0.66
0.64
0.62
0.60
0.58
input 1 2 3 4 5 6 7 8 9 10 11 12 13 14 15 16 17 18 19 RSR
frame (1000 MD steps/frame)
Clashscore
25
20
15
10
5
input 1 2 3 4 5 6 7 8 9 10 11 12 13 14 15 16 17 18 19 RSR
frame (1000 MD steps/frame)
Beta-Gal(EMD-2984 and 5A1A)
CC
0.765
0.760
0.755
0.750
0.745
0.740
0.735
input 1 2 3 4 5 6 7 8 9 10 11 12 13 14 15 16 17 18 19 RSR
frame (1000 MD steps/frame)
Clashscore
10
8
6
4
2
input 1 2 3 4 5 6 7 8 9 10 11 12 13 14 15 16 17 18 19 RSR
frame (1000 MD steps/frame)

Figure S1

Supplementary Table 1

pdb	map	res. (Å)	model type		Clashscore	Ramachandran plot Favored	Allowed	Outliers	C-beta deviations	Rotamer outliers	phenix CC	Cis-peptides	Rosetta score
5ye5	6826	5.8	CA/PA	**Input**	28.01	1720	56	32	0	22.22%	0.6750	24	80206
				**LF**	1.85	1651	85	72	135	6.39%	0.5887	23	-7124
				**LF_RSR**	4.90	1640	152	16	0	0.24%	0.6187	20	-8510
5k12	8194	1.8	FAT	**Input**	15.66	1674	66	0	0	0.82%	0.8670	6	-122915
				**LF**	1.02	1652	74	14	68	2.53%	0.8493	6	-120715
				**LF_RSR**	1.86	1716	24	0	0	0.41%	0.8202	6	-128126
6b44	7048	2.9	FAT/PA	**Input**	5.34	2528	326	0	0	0.43%	0.7664	0	-51090
				**LF**	2.48	2496	266	92	114	4.30%	0.6833	0	-92158
				**LF_RSR**	5.78	2617	236	1	1	0.13%	0.7488	0	-107532
4v1w	2788	4.7	FAT	**Input**	7.78	3889	119	24	172	7.67%	0.8161	0	-18504
				**LF**	1.17	3820	169	43	351	2.31%	0.7736	0	-21703
				**LF_RSR**	4.77	3912	120	0	0	0.00%	0.8040	0	-24099
5np0	3669	5.7	PA	**Input**	67.09	4463	393	14	0	0.00%	0.8063	0	748887
				**LF**	10.23	4254	380	238	393	7.83%	0.7697	0	-40843
				**LF_RSR**	13.74	4321	545	14	6	0.57%	0.7949	0	-54879
3jbr	6475	4.2	FAT/PA	**Input**	18.27	2067	207	172	0	0.37%	0.6931	25	63922
				**LF**	3.34	2115	200	131	154	4.33%	0.6106	25	-10028
				**LF_RSR**	9.01	2121	307	18	1	0.47%	0.6831	25	-11471
5ni1	3488	3.2	FAT	**Input**	2.97	539	27	0	0	0.43%	0.8352	0	-22631
				**LF**	0.46	540	21	5	24	2.38%	0.7858	0	-20323
				**LF_RSR**	1.71	548	18	0	0	0.00%	0.8265	0	-22862
5sy1	8315	3.9	FAT	**Input**	5.24	1341	103	2	0	0.08%	0.7808	0	-11632
				**LF**	3.37	1336	90	20	62	2.66%	0.7364	0	-13686
				**LF_RSR**	5.84	1372	72	2	0	0.00%	0.7735	0	-17823
5n9y	3605	4.2	FAT	**Input**	12.67	1490	135	0	0	2.02%	0.8259	0	-12745
				**LF**	1.72	1483	104	38	90	8.66%	0.7904	0	-11940
				**LF_RSR**	10.68	1544	81	0	0	0.72%	0.8266	0	-13580
5jlf	8162	3.6	FAT/PA	**Input**	8.79	2012	54	10	17	1.29%	0.7302	5	-30656
				**LF**	1.42	1962	79	35	125	4.98%	0.7044	5	-28221
				**LF_RSR**	4.63	1997	74	5	0	0.00%	0.7317	5	-29989
3j9c	6224	2.9	FAT	**Input**	6.07	401	20	0	0	6.13%	0.8286	1	-18521
				**LF**	0.76	390	27	4	7	3.47%	0.7890	1	-17624
				**LF_RSR**	4.70	405	16	0	0	0.27%	0.8304	1	-19775
5h64	6668	4.4	FAT/PA	**Input**	51.29	5088	1098	154	0	0.80%	0.7278	134	203740
				**LF**	3.20	5057	961	322	455	4.41%	0.7057	134	-32771
				**LF_RSR**	9.10	5606	685	49	1	0.39%	0.7305	129	-40825
5wq7	6675	3.0	FAT	**Input**	26.87	6630	285	45	15	0.49%	0.8152	15	-230152
				**LF**	1.60	6492	373	95	317	4.12%	0.7790	15	-214396
				**LF_RSR**	5.39	6701	259	0	0	0.00%	0.8182	15	-239091
6ayf	7019	3.6	FAT	**Input**	17.90	1748	128	4	0	0.11%	0.8066	0	-22033
				**LF**	1.11	1751	118	11	96	4.37%	0.7782	0	-23433
				**LF_RSR**	2.83	1798	82	0	0	0.00%	0.8105	0	-27527
5a63	3061	3.4	FAT	**Input**	39.82	1108	77	26	8	13.74%	0.8001	0	-30726
				**LF**	2.07	1095	82	34	83	5.44%	0.7557	0	-35101
				**LF_RSR**	5.80	1123	87	1	0	0.67%	0.8054	0	-39137
5a1a	2984	2.2	FAT	**Input**	11.62	3896	160	24	0	1.03%	0.7636	56	-240900
				**LF**	1.53	3841	206	33	167	3.78%	0.7364	56	-232436
				**LF_RSR**	5.51	3939	141	0	0	0.11%	0.7694	56	-251012
5kne	8276	5.6	CA	**Input**	41.62	3221	656	83	0	0.00%	0.8062	0	65030
				**LF**	3.61	3128	636	196	269	5.93%	0.7378	0	-22207
				**LF_RSR**	20.13	3437	515	8	0	0.46%	0.8122	0	-24327
5of4	3802	4.4	FAT/PA	**Input**	9.85	2079	265	15	20	1.90%	0.6930	2	-9493
				**LF**	1.59	2104	172	83	120	5.07%	0.6402	2	-15031
				**LF_RSR**	10.62	2115	244	0	0	0.40%	0.7076	2	-15839
5uj9	8559	3.5	FAT/PA	**Input**	0.65	1224	53	0	0	0.00%	0.8129	0	-42877
				**LF**	1.05	1196	63	18	69	3.92%	0.7937	0	-39510
				**LF_RSR**	3.51	1223	54	0	0	0.20%	0.8274	0	-44563
5u0p	8479	4.4	FAT/PA	**Input**	17.73	2538	216	87	1	0.00%	0.7496	42	3061
				**LF**	3.14	2508	225	108	166	4.26%	0.7543	42	-28167
				**LF_RSR**	11.35	2558	278	5	0	0.25%	0.7703	42	-29838

Supplementary Table 1 (Continued)

pdb	map	res. (Å)	model type		Clashscore	Ramachandran plot Favored	Allowed	Outliers	C-beta deviations	Rotamer outliers	phenix CC	Cis-peptides	Rosetta score
5gar	8016	6.4	CA	**Input**	0.00	5115	524	181	0	0.00%	0.7404	6	-38687
				**LF**	4.30	5064	506	246	322	6.34%	0.7670	6	-56362
				**LF_RSR**	10.37	5266	527	27	1	0.74%	0.7621	6	-63457
5uar	8461	3.7	FAT	**Input**	0.26	1086	87	1	0	1.06%	0.7837	0	-16845
				**LF**	0.94	1080	76	18	69	3.57%	0.7521	0	-14553
				**LF_RSR**	4.84	1106	67	1	0	0.19%	0.8074	0	-17206
3j7h	5995	3.2	FAT	**Input**	130.65	3972	100	8	0	12.24%	0.8210	64	21515
				**LF**	1.84	3774	235	71	204	5.98%	0.8124	64	-146830
				**LF_RSR**	5.42	3864	208	8	0	0.11%	0.8479	64	-163189
5m54	4156	8.0	FAT	**Input**	7.01	1749	62	0	0	0.00%	0.7978	2	-26988
				**LF**	1.63	1693	85	33	64	3.01%	0.7763	2	-24993
				**LF_RSR**	8.31	1745	64	2	0	0.00%	0.8306	2	-27249
6bqr	7132	3.2	FAT/PA	**Input**	25.51	3360	360	8	0	2.07%	0.7606	0	-76190
				**LF**	1.82	3375	274	79	173	6.38%	0.7529	0	-105226
				**LF_RSR**	5.03	3416	304	8	0	0.63%	0.7946	0	-122613
5vou	8722	6.4	FAT/PA	**Input**	15.37	2132	134	0	0	0.00%	0.8029	12	19334
				**LF**	4.51	2103	128	35	110	4.29%	0.7620	12	-21193
				**LF_RSR**	15.80	2079	186	1	0	0.52%	0.8383	12	-22414
5vkq	8702	3.6	FAT/PA	**Input**	7.54	5428	532	20	0	0.72%	0.7708	0	-nan
				**LF**	3.33	5340	436	196	298	4.64%	0.7610	0	-60978
				**LF_RSR**	6.90	5502	470	8	0	0.48%	0.8133	0	-76415
5oej	3790	5.7	PA	**Input**	13.40	2264	240	115	0	0.00%	0.5559	20	160946
				**LF**	4.39	2318	174	127	135	6.07%	0.4661	20	-2014
				**LF_RSR**	10.44	2308	296	15	0	0.32%	0.6002	20	-7555
5u1d	8482	4.0	FAT/PA	**Input**	0.14	1090	66	2	0	0.84%	0.7126	0	4463
				**LF**	3.06	1057	74	27	58	3.73%	0.6988	0	-10391
				**LF_RSR**	6.28	1087	70	1	0	0.10%	0.7591	0	-13015
5gw5	9541	4.6	FAT	**Input**	5.65	8025	382	7	0	0.01%	0.6374	0	-75861
				**LF**	1.11	7842	452	120	320	2.43%	0.6493	0	-64126
				**LF_RSR**	3.19	8059	339	16	0	0.03%	0.6997	0	-72841
3jac	6343	4.8	FAT/PA	**Input**	59.58	2408	132	142	3	4.17%	0.5443	10	78873
				**LF**	4.55	2404	162	116	173	5.46%	0.5659	10	-29172
				**LF_RSR**	11.01	2351	325	6	0	0.00%	0.6105	9	-30404
5ljo	4061	4.9	FAT	**Input**	4.65	1401	151	62	1	0.15%	0.7749	5	-26521
				**LF**	1.48	1412	154	48	62	3.01%	0.8023	5	-21902
				**LF_RSR**	22.93	1432	180	2	0	0.15%	0.8446	0	-21066
5l93	4015	3.9	FAT	**Input**	3.37	601	65	0	0	0.00%	0.7419	6	-8106
				**LF**	1.35	609	53	4	40	3.57%	0.7657	6	-7533
				**LF_RSR**	4.71	633	33	0	0	0.00%	0.8115	6	-9163
5fxh	3353	5.0	PA	**Input**	17.08	2730	277	14	0	0.00%	0.7489	2	150477
				**LF**	8.94	2585	297	131	215	6.70%	0.7799	2	-41193
				**LF_RSR**	14.98	2739	273	9	3	0.79%	0.8209	2	-47853
3jd8	6640	4.4	FAT/PA	**Input**	75.57	897	134	94	5	11.82%	0.6446	8	46603
				**LF**	6.33	961	111	53	70	4.07%	0.6560	8	-6565
				**LF_RSR**	9.86	986	135	4	0	0.31%	0.7292	8	-8114
3jc7	6536	4.8	FAT	**Input**	42.20	4479	424	66	7	0.45%	0.6584	195	47295
				**LF**	1.87	4307	472	196	319	5.81%	0.6867	196	-30588
				**LF_RSR**	11.57	4395	557	23	2	0.31%	0.7509	192	-34889
3jch	6553	7.1	CA	**Input**	2.28	1567	80	0	0	0.00%	0.7030	0	-20566
				**LF**	5.10	1517	79	51	87	5.49%	0.7791	0	-20090
				**LF_RSR**	14.00	1526	121	0	0	0.06%	0.8489	0	-23141
6eny	3906	5.8	PA	**Input**	12.90	1368	136	33	0	0.00%	0.5648	12	61616
				**LF**	7.94	1312	163	64	71	6.19%	0.7795	12	-12663
				**LF_RSR**	17.71	1383	154	2	0	0.60%	0.7362	12	-15692
5nd7	3623	7.9	FAT	**Input**	19.97	1024	84	10	6	14.15%	0.5818	0	-10039
				**LF**	1.57	998	89	31	62	5.91%	0.6993	0	-12488
				**LF_RSR**	8.10	1034	83	1	0	0.00%	0.7673	0	-13362
